# Transepithelial Transport and Metabolism of Boronated Dipeptides Across Caco-2 and HCT-8 Cell Monolayers

**DOI:** 10.1155/MBD.1996.277

**Published:** 1996

**Authors:** Amy L. Elkins, John G. Eley, Merrill C. Miller III, Iris H. Hall, Anup Sood, Bernard Spielvogel

**Affiliations:** 1 Division of Medicinal Chemistry and Natural Products School of Pharmacy Beard Hall, CB# 7360 University of North Carolina Chapel Hill NC 27599-7360 USA; 2 Boron Biologicals Inc. 620 Huitton St. Suite 104 Raleigh NC 27606 USA; 3 School of Pharmacy Samford University Birmingham AL 35229 USA

## Abstract

Oral delivery of proteins and peptides as therapeutic agents is problematic due to their low bioavailability. This study examined the effect of boronation on the transepithelial transport and metabolism of three glycine-phenylalanine dipeptides in Caco-2 and HCT-8 cell monolayers. The three dipeptides exhibited passive transport characteristics in the monolayer systems. However, metabolism of the boronated dipeptides did occur, but to a lesser extent than the non-boronated glycine-phenylalanine dipeptide. The same metabolic scheme was seen in both cell monolayer system, but greater metabolism was seen in the HCT-8 cell monolayers.


### TRANSEPITHELIAL TRANSPORT AND METABOLISM OF BORONATED DIPEPTIDES ACROSS CACO-2 AND HCT-8 CELL MONOLAYERS

Amy L. Elkins[1], John G. Eley[1,3], Merrill C. Miller III[1], Iris H. Hall*[1], Anup Sood[2] and Bernard Spielvogel[2]

[1] Division of Medicinal Chemistry and Natural Products, School of Pharmacy, Beard Hall, CB# 7360, University of North Carolina at Chapel Hill, NC 27599-7360, USA
[2] Boron Biologicals, Inc., 620 Huitton St., Suite 104, Raleigh, NC 27606, USA
[3] School of Pharmacy, Samford University, Birmingham, AL 35229, USA

### ABSTRACT

Oral delivery of proteins and peptides as therapeutic agents is problematic due to their low bioavailability. This study examined the effect of boronation on the transepithelial transport and metabolism of three glycine-phenylalanine dipeptides in Caco-2 and HCT-8 cell monolayers. The three dipeptides exhibited passive transport characteristics in the monolayer systems. However, metabolism of the boronated dipeptides did occur, but to a lesser extent than the non-boronated glycine-phenylalanine dipeptide. The same metabolic scheme was seen in both cell monolayer system, but greater metabolism was seen in the HCT-8 cell monolayers.

### INTRODUCTION

Boron derivatives of amino acids and di- and tripeptides have been synthesized with a boron atom incorporated in place of the carbon atom in the alkyl chain. A number of these compounds have been screened for potential activity and have been found to exhibit antineoplastic, anti-inflammatory, anti-osteoporosis, and hypolipidemic properties. One of these compounds, N-[(trimethylamine-boryl)-carbonyl]-L-phenylalanine methyl ester, *I*, exhibited potent cytotoxicity against various murine and human tissue cultured cancer cells (1). In $L_{1210}$ cells, the boron derivative blocked DNA, RNA, and protein synthesis in a concentration dependent manner achieving greater than 50% inhibition at 100 μM (1). From these results, it is evident that *I* is able to penetrate cellular barriers to inhibit cell growth.

Oral delivery of proteins and peptides as therapeutic agents is problematic due to their low bioavailability. This is mainly due to both their poor permeability across cellular barriers and to enzymatic degradation that occurs in the gut lumen, brush border, and enterocyte cytoplasm (2). Substitution of a boron atom for an amino α-carbon in *I* demonstrated an increased *in vitro* stability (3). Such as a substitution may also have the potential to alter the *in vivo* bioavailability of dipeptides. These characteristics could have beneficial therapeutic applications in the development of peptidyl drug therapies.

This study was undertaken to determine the effect of boronation on the transepithelial transport and metabolism of three glycine-phenylalanine dipeptides. The transport and metabolism of two boron substituted dipeptides, *I* and N-[(dimethylamine-boryl)-carbonyl]-L-phenylalanine methyl ester, *II*, were compared to a non-boronated dipeptide, *N,N*-dimethylglycyl-L-phenylalanine methyl ester, *III*, using Caco-2 and HCT-8 cell monolayers as model systems for the small intestine epithelium (see Figure 1).

Caco-2 cells are human colon adenocarcinoma cells first isolated from a primary colonic tumor (4). The cell line undergoes spontaneous enterocytic differentiation in culture after reaching confluence, and morphologically resembles small intestinal epithelium after three weeks in culture (5). These morphological characteristics include the development of a brush border region expressing microvilli, occluding junctions, dome formations, and the formation of tight junctions leading to cell polarity. In addition, the Caco-2 cells exhibit small intestine biochemical markers such as brush border enzymes, and enzymes are secreted into the growth medium from both the apical and basal regions (6-8). The cell line expresses a dipeptide carrier transport system in both the apical and basal membranes (9-10). Caco-2 cells also exhibit transport pathways similar to the distal ileum, and the electrical properties are closely related to colonic epithelium.

HCT-8 cells are human adenocarcinoma cells from the ileocecal region; as such, these cells may give a truer reflection of gut absorption compared to Caco-2 cells which are derived from the colonic region. The cells produce a polarized differentiated monolayer consisting of enterocyte-like columnar cells that are interconnected by mature tight junctions and desmosomes and exposed microvilla (11). The structural and functional integrity of the tight junctions of cells grown on polyester filter cups (Falcon, BD) and membrane filter inserts (Transwell®, Costar) have been confirmed by transmission electron microscopy, transepithelial electrical resistance measurements, and transport of impermeant markers such as mannitol and polyethylene glycol (11, 12).

### METHODS

### Sources of Compounds

The synthesis of *I* has been reported (13). Compound *II* was donated by Boron Biologicals, Inc., Raleigh, NC. Compound *III* was synthesized as follows: L-phenylalanine methyl ester hydrochloride (2.09 g, 9.7 mmol) and dimethylglycine (1 g, 9.7 mmol) were suspended in 30 ml dry tetrahydrofuran with 10 mmol of triethylamine, dicyclohexyl-carbodiimide and hydroxybenzotriazole. The reaction was stirred at room temperature for 48 hours under nitrogen and the solvent was removed under reduced pressure. The product was purified on silica gel, two void volumes of hexane, two void volumes hexane/ethanol (8:1), and then hexane/ethanol (5:1). The combined fractions of purified product yielded a clear liquid (421.9 mg, 16.7%), and had a $R_f = 0.35$ using 1:1 hexane/ethanol. $^1H$ NMR ($CDCl_3$) δ 7.55 (d, 1H, NH), δ 7.26 (m, 5H, $C_6H_5$), δ 4.91 (m, 1H, CH), δ 3.73 (s, 3H, $CH_3$), δ 3.18 (o, 2H, $CH_2$), δ 2.92 (d, 2H, $CH_2$), δ 2.20 (s, 6H, $CH_3$). The elemental analysis for $C_{14}H_{20}O_3$ (F.W. = 264.32 g/mol) was calculated to be C, 63.62%, H, 7.63%, and N, 10.60%. The elemental analysis found C, 63.82%, H, 7.60%, and N, 10.57%.

Figure 1 Structures of Compounds

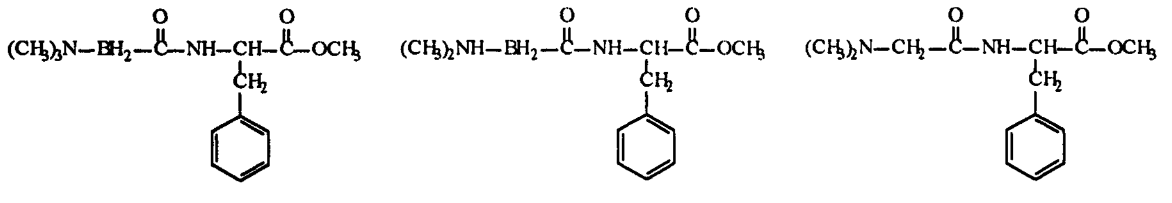

| *I* | *II* | *III* |
|---|---|---|
| N-[trimethylamine-boryl)-carbonyl]-L-phenylalanine methyl ester | N-[(dimethylamine-boryl)-carbonyl]-L-phenylalanine methyl ester | N,N-dimethylglycyl-L-phenylalanine methyl ester |

### Cell Lines

Caco-2

The Caco-2 cell line was obtained from the Lineberger Cancer Center at the University of North Carolina at Chapel Hill at different serial passage numbers (13-16). For the transport studies, cells were diluted with growth medium to give a total of $7.5 \times 10^4$ cells/cm$^2$ in 1.5 mls. The 1.5 mls was placed in the apical compartment of a Transwell® (Costar). The Transwell® system is a 6-well plate with each well having an apical compartment capacity of 1.5 mls, a basal compartment capacity of 2.5 mls, and a 4.71 cm$^2$ polycarbonate microporous (0.45 μm) membrane separating the two compartments. Cell monolayers in the Transwell® were allowed to grow for 21 days while the medium was replaced every 3 days. The cells were grown in Dulbecco's Modified Eagle Medium (D-MEM) containing L-glutamine and sodium pyruvate at 37°C under 5% $CO_2$ and saturated humidity. Additives to the medium included 10% heat-inactivated fetal bovine serum, 10 mM nonessential amino acids, 100 units of penicillin and 100 mg of streptomycin per ml of medium.

On the day of the transport study, the culture was placed in a medium of Hank's Balanced Salt Solution (HBSS) (Gibco) containing 0.35 g sodium bicarbonate and 1.25 g *N*-(2-hydroxyethyl)-piperazine-*N'*-2-ethanesulfonic acid (HEPES) (Sigma Chemical Co.) and allowed to equilibrate for 1 hour. The apical-to-basal transport experiment was initiated by removing the buffer solution from the apical compartment and adding 1.5 ml of fresh HBSS/HEPES containing the compound. At specified time intervals (0.25, 0.50, 1.0, 1.5, 2.0, 3.0, and 4.0 hours), the monolayer insert (the apical compartment with membrane) was moved to a new well with the basal compartment containing 2.5 ml fresh HBSS/HEPES buffer to maintain sink conditions. Basolateral solutions at each time interval were collected and analyzed by HPLC (see below). After 4 hours, the remaining apical solution was collected for HPLC analysis. All experiments at 37°C were performed in an incubator at 5% $CO_2$ and 95% atmosphere. Experiments conducted at 4°C were performed in a cold room. All experiments were performed in duplicate; when agitation was desired, the Transwell® system was placed on a cell Nutator (Clay Adams, Model 1105).
The monolayer integrity was tested both before and after transport experiments by monitoring the passage of the fluid-phase marker Lucifer yellow CH from the apical to the basal compartment. The integrity was further tested by measuring the potential difference across the monolayer using a transepithelial electrical resistance device described elsewhere (14) in which 50 μA of current is applied across the monolayer and the electrical resistance across tight junctions is determined from Ohm's Law in units of $\Omega \times cm^2$.
After completing the transport experiments, the monolayers were immersed in a 37% formaldehyde solution for 30 minutes. The monolayers were then rinsed in distilled water and stained for 2 minutes with Mayer's hematoxylin which has an affinity for negatively charged particles and reveals DNA, RNA, and acidic proteins. After a second rinsing with distilled water, the monolayers were covered with an eosin-phloxine solution (87% ethanol, 11% eosin, 1% phloxine, and 0.4% glacial acetic acid) which stained cellular proteins. Areas devoid of growth where leakage may have occurred were not stained and such data were not included in analyses.

HCT-8
The HCT-8 cell line was obtained from the Lineberger Cancer Center at the University of North Carolina at Chapel Hill at serial passage number 190. The transport experiment protocol was the same as described in the Caco-2 cell experiments.

**Partition Coefficient**
The apparent partition coefficient (PC') was obtained by initially preparing 2 mM solutions of compounds *I-III* in HBSS/HEPES buffer. One milliliter of water saturated n-octanol and 1 ml of the buffer were added to 5 ml vials for duplicate determinations. Each vial was mixed for 2 minutes with a vortex mixer at room temperature, and then centrifuged at 2000 rpm for 10 minutes. The n-octanol layer was removed using a disposable pipet, and the remaining n-octanol film was removed by vacuum suction. HPLC analysis (see below) was used to determine the concentration of the compounds.

**HPLC Analysis**
For preliminary transport studies (Tables 1 - 8 in the Results section), HPLC analyses for the compounds were performed with an isocratic system consisting of a LC pump (Spectra-Physics), an autosampler (SSI, model 280D), a Dynamax UV/VIS absorbance detector (Rainin) at 203 nm, an integrator (Shimadzu, model CR601), and an Econosil $C_{18}$ 10μ (Alltech) 4.5 x 250 mm column. The mobile phase was methanol and disodium phosphate (60:40) at pH = 7.0. However, this initial mobile phase was altered for later studies (Table 9) to a more acidic pH (2.7) in order to lengthen the elution time of the degradation products. These later studies were performed with an isocratic system consisting of a Model 6000A pump (Waters), and Model 210B autosampler (Waters), a Dynamax UV/VIS absorbance detector (Rainin) set at 210 nm, a Econosil $C_{18}$ 10m column (Alltech), and a model CR601 integrator (Shimadzu). The flow rate was 1.5 ml/min and the k' values for compounds *I-III* were 2.3, 2.5, and 2.8 respectively. The following mobile phases were used for each compound: *I* and *II*, methanol/0.25% $H_3PO_4$ in a 60/40 ratio at pH 2.7; and *III*, methanol/0.1N $NaH_2PO_4$ in a 60/40 ratio at pH 7.0. The respective free acids of the three compounds (see Results) were detectable without modifying the appropriate mobile phases. All quantitations were accomplished using standard calibration curves of the individual compounds.

### RESULTS

The stability of the three compounds was determined in buffer and media without the Transwell® system. Each of the three compounds were found to not degrade in HBSS/HEPES buffer solution at pH 7.4 and 37°C, or the apical and basal media for 10 hours. When the study was extended, degradation was noted only in the last sample taken at 25 hours. At 25 hours, *I* contained 96% of the original ester, *II* contained 90%, and *III* contained only 55%. These results show that the three compounds undergo some degradation between 10 and 25 hours. However, the transport experiment was conducted for only 4 hours, and therefore hydrolysis in buffer solution at pH 7.4 and 37°C should not be a factor.

To determine if transport of *I* was concentration dependent, varying concentrations of the compound was studied. Table 1 shows that the mass of *I* transported across Caco-2 cell monolayers was proportional to the initial concentration over the range of 0.75 to 7.50 mM. Essentially the same percentage of mass was transported at each concentration. These results suggest that *I* was transported across the Caco-2 cell monolayer by passive diffusion. A plot of flux versus initial concentration was linear (plot not shown), again indicating that passive transport was occurring.

Table 1 Averaged Cumulative Recovery of Compound *I* After Four Hours of Mass Transport Across Caco-2 Cell Monolayers at 37°C and Static Conditions (n = 2).

| Initial Concentration[a] | Mass Transported[b] | % Mass Transported | % Mass in Apical Side | % Mass Balance |
|---|---|---|---|---|
| 0.75 | 0.70 | 62.2 | 1.6 | 63.8 |
| 1.0 | 0.90 | 60.0 | 7.8 | 67.8 |
| 2.5 | 2.4 | 64.0 | 9.0 | 73.0 |
| 5.0 | 4.7 | 62.7 | 8.3 | 71.0 |
| 7.5 | 7.5 | 66.7 | 7.9 | 74.6 |

[a] initial concentration of *I* in mM.
[b] mmol collected in the basal medium after 4 hours.

The transport of *I* followed first-order kinetics, with mass transported increasing with time. The following equation was fitted to the experimental data using nonlinear least-squares analysis where $\beta_t$ and $\beta_\infty$ equal the amount of *I* in the basal compartment at times t and $\infty$ respectively, and k equals the apparent first-order rate constant.

$$\beta_t = \beta_\infty (1 - e^{-kt})$$

The apparent permeability coefficient (Pe) can be calculated from the slope of a plot of the mass transported divided by the product of the Transwell® surface area (4.71 $cm^2$) and the initial concentration versus time (14). Alternatively, Pe can be calculated from the derivative of the first order equation at time zero, e.g., k x $\beta_\infty$. The rate constants (k) and permeability coefficients (Pe) for the transport experiments using *I* at different initial concentrations are given in Table 2. The values of k and Pe for the various initial concentrations were not significantly different, again indicating that passive diffusion was occurring.

Table 2 The Pseudo First-Order Rate Constants (k) and the Apparent Permeability Coefficients (Pe) for Four Hours of Mass Transport of Compound *I* Across Caco-2 Cell Monolayers at 37°C and Static Conditions (n = 2).

| Initial Concentration[a] | k ± SD x $10^4$/$sec^{-1}$ | Pe ± SD x $10^{-5}$ cm/sec |
|---|---|---|
| 0.75 | 1.76 ± 0.06 | 3.95 ± 0.16 |
| 1.0 | 1.70 ± 0.08 | 3.51 ± 0.18 |
| 2.5 | 1.63 ± 0.08 | 3.75 ± 0.21 |
| 5.0 | 1.35 ± 0.17 | 3.20 ± 0.37 |
| 7.5 | 1.56 ± 0.11 | 3.75 ± 0.28 |

[a] initial concentration of *I* in mM.

Experiments performed by agitating (rocking) the cell monolayer on a cell Nutator showed an increase in the amount and rate of *I* transported at 37°C (see Tables 3 and 4). More of *I* remained in the apical compartment under static conditions than in rocking conditions (16% compared to 2%). At 4°C, rocking conditions, both the amount and rate of *I* transported was decreased compared to 37°C, rocking conditions.

Table 3 Averaged Cumulative Recovery of Compound *I*[a] After Four Hours of Mass Transport Across Caco-2 Cell Monolayers Under Varying Conditions (n = 2).

| | Mass Transported[b] | % Mass Transported | % Mass in Apical Side | % Mass Balance |
|---|---|---|---|---|
| 37°C Static | 2.1 | 56.9 | 16.2 | 73.1 |
| 37°C Rocking | 3.0 | 78.5 | 0.80 | 79.3 |
| 4°C Rocking | 1.8 | 48.6 | 40.7 | 89.3 |

[a] initial concentration of *I* was 2.5 mM. [b] mmol collected in the basal medium after 4 hours.

Table 4 The Pseudo First-Order Rate Constants (k) and the Apparent Permeability Coefficients (Pe) for Four Hours of Mass Transport of Compound *I*[a] Across Caco-2 Cell Monolayers Under Varying Conditions (n = 2).

| | $k \pm SD \times 10^4/sec^{-1}$ | $Pe \pm SD \times 10^{-5}$ cm/sec |
|---|---|---|
| 37°C Static | 1.03 ± 0.06 | 2.36 ± 0.14 |
| 37°C Rocking | 3.54 ± 0.06 | 8.87 ± 0.24 |
| 4°C Rocking | 1.44 ± 0.19 | 2.47 ± 0.47 |

[a] initial concentration of *I* was 2.5 mM.

Compound *II* was found to transport through the cell monolayers by passive diffusion using the same criteria applied to *I* (data not shown). At 37°C, rocking conditions, the rate constant (k) and permeability (Pe) of II were significantly less than the values for *I* and *III* (Tables 5 - 8). However, *II* degrades at a faster rate than I, but not as quickly as *III* when exposed to Caco-2 cell monolayers. Degradation was reduced at 4°C but not completely inhibited. The permeability values at 4°C were not significantly different between rocking and static conditions.

Table 5 Averaged Cumulative Recovery of Compound *II*[a] After Four Hours of Mass Transport Across Caco-2 Cell Monolayers Under Varying Conditions (n = 2).

| | Mass Transported[b] | % Mass Transported | % Mass in Apical Side | % Mass Balance |
|---|---|---|---|---|
| 37°C Static | 0.79 | 52.7 | 5.0 | 57.7 |
| 4°C Static | 0.59 | 39.3 | 40.6 | 79.9 |
| 4°C Rocking | 0.56 | 37.3 | 44.5 | 81.8 |

[a] initial concentration of *II* was 1 mM. [b] mmol collected in the basal medium after 4 hours.

Table 6 The Pseudo First-Order Rate Constants (k) and the Apparent Permeability Coefficients (Pe) for Four Hours of Mass Transport of Compound *II*[a] Across Caco-2 Cell Monolayers Under Varying Conditions (n = 2).

| | $k \pm SD \times 10^4/sec^{-1}$ | $Pe \pm SD \times 10^{-5}$ cm/sec |
|---|---|---|
| 37°C Rocking | 2.8 ± 0.22 | 4.8 ± 0.48 |
| 4°C Static | 0.8 ± 0.11 | 1.4 ± 0.41 |
| 4°C Rocking | 0.9 ± 0.11 | 1.4 ± 0.28 |

[a] initial concentration of *II* was 1 mM.

Table 7 Averaged Cumulative Recovery of Compound *III*[a] After Four Hours of Mass Transport Across Caco-2 Cell Monolayers Under Varying Conditions (n = 2).

| | Mass Transported[b] | % Mass Transported | % Mass in Apical Side | % Mass Balance |
|---|---|---|---|---|
| 37°C Static | 0.38 | 25.3 | 16.2 | 41.5 |
| 37°C Rocking | 0.56 | 37.3 | 14.0 | 51.3 |
| 4°C Static | 0.28 | 18.7 | 63.8 | 82.5 |
| 4°C Rocking | 0.65 | 43.3 | 24.8 | 68.1 |

[a] initial concentration of *III* was 1 mM. [b] mmol collected in the basal medium after 4 hours.

Table 8 The Pseudo First-Order Rate Constants (k) and the Apparent Permeability Coefficients (Pe) for Four Hours of Mass Transport of Compound *III*[a] Across Caco-2 Cell Monolayers Under Varying Conditions (n = 2).

| | k ± SD x $10^4$/sec$^{-1}$ | Pe ± SD x $10^{-5}$ cm/sec |
|---|---|---|
| 37°C Rocking | 8.5 ± 0.14 | 10.7 ± 0.41 |
| 4°C Static | 1.0 ± 0.08 | 0.8 ± 0.12 |
| 4°C Rocking | 1.5 ± 0.06 | 2.2 ± 0.12 |

[a] initial concentration of *I* was 1 mM.

Compound *III* also was found to transport through the monolayers via passive diffusion (data not shown). At 37°C, rocking conditions, the rate constant (k) and permeability (Pe) of *III* were significantly greater than the values for *I* and *II* (Tables 7 and 8). However, *III* degrades at a faster rate than the other two compounds when exposed to the cell monolayers. Degradation was reduced at 4°C but not completely inhibited; however, the permeability values at 4°C were significantly different between rocking and static conditions (Student's t-test, 95% confidence).

Approximately 70% of Compound *I* was recovered after 4 hours (see Table 1). During those experiments, one additional peak was seen to elute to earlier than *I*. To determine if this degradation product was the result of ester hydrolysis, I was incubated with 0.01N NaOH at 37°C for 6 hours. HPLC conditions were modified as described in the Methods section and the analysis of the incubated solution showed a peak that occurred at the same retention time as the degradation product observed in the transport experiments. A time study of the incubation solution was done by injecting numerous samples over a 6 hour period, and the ester peak continued to decrease with a simultaneous increase in the degradation peak. After 6 hours, all of the ester was degraded to what was thought to be the free acid of *I*. To further confirm the identity of the degradation peak, *II* and phenylalanine methyl ester were analyzed by HPLC; both compounds had a different retention time than both *I* and the presumed free acid peaks. The identity was finally confirmed as the free acid of *I* by NMR. Identical studies were conducted with compounds *II* and *III*; and their free acids confirmed.

Initial experiments with *I* showed that transport across Caco-2 cell monolayers occurred by passive diffusion. Further, the initial experiments showed that the Caco-2 cell monolayers had some metabolic specificity for all three compounds. A set of experiments were designed to compare the effects of boronation on transport and metabolism. Side-by-side experiments were conducted with Caco-2 cell monolayers to ensure that the enzyme concentration was as comparable as possible for each compound studied. Experiments were performed in duplicate using 1 mM of Compounds *I-III*.

At 37°C, static conditions, 44% of *I* was transported over four hours (see Table 9), and none of *I* remained in the apical compartment; only the free acid of *I* was found in the apical compartment. In the side-by-side experiment, 44% of I was found as the free acid in the basal compartment, confirming that I was being substantially degraded as it transported through the cell monolayer. At 4°C, at both static and rocking conditions, degradation of *I* to its free acid was inhibited since none of the free acid was found in the basal compartment or remained in the apical compartment. The transport profile of *II* was similar to *I*. After 4 hours of transport at 37°C, static conditions, 49% of *II* was recovered in the basal compartment with no remaining ester present in the apical compartment. In the side-by-side experiment, 52% of *II* was degraded to the corresponding free acid as *II* transported through the monolayer. At 4°C, at either static or rocking conditions, the degradation of *II* to its respective free acid was inhibited, again, similar to Compound *I*. The transport profile of compound *III* was similar to *I* and *II*; however, *III* differed from *I* and *II* in that degradation was not completely inhibited at 4°C. But it was markedly decreased compared to the 37°C, static conditions.

Table 9 Averaged Cumulative Recovery of Compounds *I-III*[a] and Their Corresponding Free Acids In the Basal Compartment After Mass Transport for Four Hours Across Caco-2 Cell Monolayers Under Varying Conditions (n = 2).

| Conditions | % Ester | % FA |
|---|---|---|
| Compound *I* | | |
| 37°C Static | 44.4 | 44.4 |
| 4°C Static | 61.8 | - |
| 4°C Rocking | 74.0 | - |
| Compound *II* | | |
| 37°C Static | 48.6 | 51.5 |
| 4°C Static | 59.4 | - |
| 4°C Rocking | 55.5 | - |
| Compound *II* | | |
| 37°C Static | 38.1 | 45.7 |
| 4°C Static | 33.2 | 3.0 |
| 4°C Rocking | 64.6 | 10.6 |

[a] initial concentration of compounds was 1 mM.

Table 10 Averaged Cumulative Recovery of Compounds *I* and *III* After Four Hours of Mass Transport Across HCT-8 Cell Monolayers at 37°C, and Static Conditions (n = 2).

| Initial Concentration[a] | Mass Transported[b] | % Mass Transported | % Mass in Apical Side | % Mass Balance |
|---|---|---|---|---|
| Compound *I* | | | | |
| 0.75 | 0.87 | 77.3 | 10.9 | 88.2 |
| 1.5 | 1.02 | 45.3 | 5.3 | 50.3 |
| Compound *III* | | | | |
| 0.75 | 0.058 | 5.2 | 0.5 | 5.7 |
| 1.5 | 0.077 | 3.4 | 0.3 | 3.7 |

[a] initial concentration of compounds in mM. [b] μM collected in the basal medium after 4 hours.

Table 11 Averaged Apparent Permeability Coefficients (Pe) for Four Hours of Mass Transport of Compounds *I* and *III* Across HCT-8 Cell Monolayers at 37°C and Static Conditions (n = 2).

| Initial Concentration[a] | Pe (cm/sec) |
|---|---|
| Compound *I* | |
| 0.75 | $1.6 \times 10^{-5}$ |
| 1.5 | $1.1 \times 10^{-5}$ |
| Compound *III* | |
| 0.75 | $5.5 \times 10^{-7}$ |
| 1.5 | $2.2 \times 10^{-6}$ |

[a] initial concentrations of compounds in mM.

Table 12 Apparent Partition Coefficients (PC') of Compounds *I* - *III*[a] Between n-Octanol and HBSS/HEPES Buffer at 25°C at pH 7.4.

| Compound | log PC' |
|---|---|
| *I* | 0.66 |
| *II* | 0.61 |
| *III* | 1.12 |

[a] initial concentration of compounds was 2 mM.

In the HCT-8 cell monolayer system, compounds *I* and *III* transport was studied at only two concentrations, 0.75 mM and 1.5 mM (see Table 10). With two data points per experiment, it is more difficult to conclusively determine that Compounds *I* and *III* are transported through HCT-8 cell monolayers by passive diffusion using the same criteria applied to the Caco-2 experiments. The apparent permeability coefficients of *I* showed little change as the initial concentration changed (see Table 11), again supporting the hypothesis that *I* transports by passive diffusion through HCT-8 cell monolayers. However, Pe was greater for the higher initial concentration of Compound *III*.

Approximately 95% of *III* and 45% of *I* were unaccounted for as the ester or their respective free acid. These data suggest that in addition to a ester hydrolysis pathway, other metabolic pathways were active in the HCT-8 cell monolayers. One possibility is that the compounds underwent amide hydrolysis; however, no L-phenylalanine methyl ester was detected by HPLC.

Apparent partition coefficients (PC') were determined for all three compounds and are presented in Table 12. Compound *III* had the highest PC', meaning that compared to the other two compounds, a greater amount of *III* was found in n-octanol than in buffer. Compound *III*, the non-boronated compound, was therefore more lipophilic and the boronated compounds were more hydrophilic.

### DISCUSSION

The first aim of the study was to characterize the transepithelial transport of two boronated dipeptides and compare their transport properties with a structurally related, non-boronated, dipeptide analog. Compound *I* is a glycine-phenylalanine derivative with boron substituted at the α-carbon position of glycine. The N-terminal position contains three methyl groups resulting in an increase in lipophilicity. Likewise, the C-terminal end is blocked by a methyl ester. The partial positive charge surrounding the N-terminal nitrogen is offset by the partial negative charge from boron so that the compound should remain uncharged at physiological pH. Compound *II* is another boron substituted glycine-phenylalanine analog with the boron substitution occurring at the α-carbon position of glycine as in *I*. Two methyl groups surround the N-terminal nitrogen and the C-terminal position is again blocked by a methyl ester. Compound *III* is a dipeptide analog of *I* without boron substitution. In this compound, the N-terminal is blocked by two methyl groups to afford enhanced lipophilicity and stability without introducing a charge on the nitrogen. The C-terminal position is blocked by a methyl ester as in *I*.

The conclusion that Compounds *I-III* undergo transepithelial transport in Caco-2 cell monolayers via a passive diffusional process was supported by the following evidence. Mass transported across the cell monolayer is proportional to the initial concentration in the apical compartment over a concentration range of 0.75 to 7.50 mM (Compound *I*, see Table 1; Compounds *II* and *III*, data not shown). The apparent permeability coefficients obtained from the transport data for each initial concentration are not significantly different except Table 2 instead of Table 1. The transport of *I* and *III* across HCT-8 cell monolayers may be via passive transport, it is difficult to substantiate using only two concentrations (see Tables 10 and 11).

According to Fick's Law, decreasing the contribution of the aqueous boundary layer to transport by agitation should increase the rate of transport for lipophilic compounds for which the aqueous boundary layer is a rate determining step. In accordance with this expectation, *I* transport was significantly increased as judged by percent mass transported and the apparent permeability coefficient when the system was agitated at 37°C (see Table 3 and 4). Similar experiments were not conducted with Compounds *II* and *III* at 37°C, rocking conditions.

Temperature dependent studies were designed to show significant differences in transport rates between experiments performed at 37°C and 4°C. Active processes that contribute to transport should be inhibited at 4°C and therefore the rate and permeability values should be much higher at 37°C compared to 4°C. Both transport rates and permeability values were higher at 37°C for all three compounds as expected.

The second aim of the study was to compare the metabolism of the three peptides during transepithelial transport. Dipeptides with glycine as N-terminal residues have been shown to have a slower rate of hydrolysis than other dipeptides due to the decreased enzymatic affinity towards glycine. However, various studies performed with glycine-phenylalanine have shown that the dipeptide is extensively metabolized by enzymes *in vivo*. Two human perfusion studies, perfusing glycine-phenylalanine into the jejunum, have shown a 68% and 79% disappearance rate respectively (16, 17). Glycine-phenylalanine has also been shown to be a substrate for brush border aminopeptidases in the rat with an 85% substrate specificity (18). Possible sites for enzymatic degradation in the three compounds chosen for the present study include the amide bond and the methyl ester on the C-terminal portion of the molecule. However, the addition of blocking groups on both ends of the peptide derivatives should afford increased stability towards enzymatic degradation during transport studies.

When *I* transported through the Caco-2 cell monolayers, approximately 70% of the compound was recovered (see Tables 1 and 3). Propyl paraben was used as a marker to test for recovery since it undergoes passive diffusion without chemical degradation. Propyl paraben transported across the Caco-2 cell monolayers by passive diffusion and with 100% recovery. Therefore, *I* was being metabolized at some point during transport. Stability experiments showed that no degradation was occurring over the 4 hour period in the medium, indicating that hydrolysis must be occurring within the cell monolayer. A series of basal-to-apical transport experiments showed that both *I* and propyl paraben were 100% recovered. Thus, the metabolism must occur in the brush border region where the *I* contacts the cell monolayer in the apical-to-basal experiments.

Similar results were found with Compounds *II* and *III* with 58% of *II* recovered (see Table 5) and 42% of *III* recovered (see Table 7). The stability studies in media also showed that Compounds *II* and *III* were stable throughout the time frame of the experiment. Basal-to-apical studies were not conducted with Compounds *II* and *III*; however, it was anticipated that similar results would be obtained as with Compound *I*. Thus the metabolism of *II* and *III* is likewise assumed to occur in the brush border region of the cell monolayer. A number of hydrolases are present in the Caco-2 cell monolayer brush border including sucrose-isomaltase, aminopeptidase N, dipeptidyl peptidase IV, alkaline phosphatase, g-glutamyl transpeptidase, lactase, trehalase, maltase, and ornithine decarboxylase (6-8).

The transport of Compounds *I* and *III* showed similar results in HCT-8 cell monolayers compared to Caco-2 cell monolayers in that *I* had much higher percent mass balances and apparent permeability coefficients compared to *III* (see Tables 1, 2, 7, 10, and 11). Although the rocking conditions, temperature conditions, propyl paraben and basal-to-apical transport studies were not conducted in HCT-8 cell monolayers, it is likely that Compounds *I* and *III* are being metabolized by the HCT-8 cell monolayer brush border enzymes. Alkaline phosphatase, leucine aminopeptidase and $Zn^{2+}$-resistant a-glucosidase have been identified in the cell line (19).

Methyl ester degradation of *III* was significantly higher than *I* in both Caco-2 and HCT-8 cell monolayers and in the buffer hydrolysis studies. The difference in hydrolysis is likely a consequence of intramolecular catalysis by the secondary amino group in the dipeptide which is absent in *I*. The protonated secondary amino group may stabilize the negative charge being developed in the transition state leading to the methyl ester hydrolysis. Such a mechanism would be absent in the boron containing N-terminus.

The data further suggest that metabolism may be partially blocked by the presence of the boron atom. Whether the boron atom inhibits enzymatic activity or is just more stable to enzyme attack remains to be elucidated. Compound *III* has only two methyl groups on the N terminal end of glycine whereas *I* has three. Steric interference may therefore be a simple explanation of Compound *I*'s increased stability.

Partition coefficients can be used to estimate the lipophilicity difference between different compounds and indicate the ability of the compounds to partition into a cell membrane. In this case, the structural differences between the three analogs are reflected in their partition coefficients which may explain the differences in transport rate and permeability (see Table 12). For example, the boron substituted analogs have a lower partition coefficient or are more hydrophilic than the non-boronated *III*. The only difference between the compounds is the additional methyl group at the N-terminal position of *I* which attributes a slight increase in partition coefficient in *I* compared to *II*.

The three glycine-phenylalanine derivatives in this study were all found to undergo passive diffusional transport in the apical-to-basal direction in Caco-2 cell monolayers. The rate of permeability appeared to follow the order of partition coefficient for the three compounds. For example, *III*, with the largest partition coefficient, transported across the Caco-2 monolayer at the fastest rate. In addition, with the partition coefficient of 1.1, the permeability of *III* is probably independent of the partition coefficient and the rate determining step for diffusion is the aqueous boundary layer adjacent to the cell surface (20). In fact, experiments performed under both static and rocking conditions show a pronounced increase in transport rate with agitation compared to static conditions. However, for the boron substituted derivatives, their partition coefficient values are very similar (0.66 and 0.61). Therefore, due to their more hydrophilic nature, partitioning into and diffusion across the cell membrane appears to be the rate determining step for transport. Examination of transport under rocking and static conditions at 4°C shows that there is virtually no difference between transport rate and permeability at the two conditions.

In summary, the boron-containing dipeptides *I* and *II* undergo transepithelial transport across the two cell lines via a passive diffusional process. Since the N-terminus is no longer a nucleophile, the dipeptides *I* and *II* were much more stable in terms of alkyl ester hydrolysis at the C-terminus than *III*. At the conclusion of transport experiments, 100% recovery of *I* and *II* was achieved after quantitating their free acids but 100% recovery of *III* was not achieved, indicating that further metabolism may be occurring at the amide bond. Even at 4°C, *III* degraded to some extent to its corresponding free acid, while *I* and *II* showed no metabolism.

It is still unclear why boron substitution at the α-carbon position in glycine should affect the stability of the phenylalanine methyl ester. This increased stability could result from a decreased affinity for the hydrolases in the brush border, or from the lower rate of permeability into the cell membrane leading to decreased exposure to the hydrolases.

ACKNOWLEDGMENTS

The studies on the Caco-2 cells were a part of Amy Elkins' thesis work under the direction of Dr. Moo Cho. The authors wish to thank Dr. Robert P. Schrewsbury for his valuable assistance with the writing of the manuscript.

REFERENCES

1. Hall IH, Hall ES, Miller III MC, Sood A, Spielvogel BF. *Amino Acids* 1993; 4:287-302.
2. Bai JPF, and Amidon GL. *Pharm Res* 1992; 9:969-78.
3. Elkins AL. Transepithelial transport of two boronated dipeptides across the Caco-2 cell monolayer. *M.Sc. Thesis UNC-CH* 1993. pp 1-78.
4. Fogh J. *J Nat Cancer Inst* 1977; 58:209-14.
5. Hidalgo IJ, Raub TJ, Borchardt RT. *Gastroent* 1989; 96:736-49.
6. Gstraunthaler G. *Renal Physiol* 1988; 11:1-42.
7. D'Agostino L, Daniele B, Pignata RG, *et al. Gastroent* 1989; 97:889-93.
8. Kenny JA, Howell S, Turner AJ. *Biochem J* 1992; 284:595-601.
9. Dantiz A, and Bergin L. *Biochim Biophys Acta* 1990; 1045:147-55.
10. Inui K, Yamamoto M, Saito H. *J Pharmacol Exp Ther* 1992; 261:195-201.
11. Zacherl J, Hamilton G, Thalhammer T, *et al. Can Chem Pharm* 1994; 34:125-32.
12. Dias VC, and Yatscoff RW. *Clin Biochem* 1994; 27:31-6.
13. Sood A, Sood CK, Spielvogel BF, Hall IH. *Eur J Med Chem* 1990; 25:301-08.
14. Cho MJ, Adson A, and Kezdy FJ. *Pharm Res* 1990; 7:325-32.
15. Lide. *Handbook of chemistry and physics*. 75th ed. Boca Raton: CRC Press, 1994.
16. Nutzenadel W, Fahr K, Lutz P. *Pediatr Res* 1981; 15:309-12.
17. Steinhardt JH, and Adibi SA. *Gastroent* 1986; 90:577-82.
18. Adibi SA, and Kim YS. Peptide absorption and hydrolysis. In: Johnson L, Ed. *Physiology of the gastrointestinal tract*. Vol. 2, Chapter 43, pp. 1082. New York: Raven Press, 1981.
19. Allen CN, Harpur ES, Bray TJB and Hirst BH. *Toxic in Vitro* 1991; 5:183-91.
20. Burton PS, Conradi RA, and Hilgers AR. *Adv Drug Deliv Rev* 1990; 7:365-86.

**Received: December 29, 1996 - Received in revised camera-ready format: January 9, 1997**

